# The *Kandelia obovata* transcription factor *KoWRKY40* enhances cold tolerance in transgenic *Arabidopsis*

**DOI:** 10.1186/s12870-022-03661-2

**Published:** 2022-06-04

**Authors:** Jiao Fei, You-Shao Wang, Hao Cheng, Yu-Bin Su, Yong-Jia Zhong, Lei Zheng

**Affiliations:** 1 State Key Laboratory of Tropical Oceanography, South China Sea Institute of Oceanology, Chinese Academy of Sciences, Guangzhou, 510301 China; 2 Southern Marine Science and Engineering Guangdong Laboratory (Guangzhou), Guangzhou, 511458 China; 3 Innovation Academy of South China Sea Ecology and Environmental Engineering, Chinese Academy of Sciences, Guangzhou, 510301 China; 4 Department of Cell Biology & Institute of Biomedicine, National Engineering Research Center of Genetic Medicine, MOE Key Laboratory of Tumor Molecular Biology, Guangdong Provincial Key Laboratory of Bioengineering Medicine, College of Life Science and Technology, Jinan University, Guangzhou, 510632 China; 5 Root Biology Center, Fujian Agriculture and Forestry University, Fuzhou, 350002 China

**Keywords:** Mangrove plant, *Kandelia obovata*, *WRKY* gene, Transgenic, Cold stress

## Abstract

**Background:**

WRKY transcription factors play key roles in plant development processes and stress response. *Kandelia obovata* is the most cold-resistant species of mangrove plants, which are the important contributors to coastal marine environment. However, there is little known about the WRKY genes in *K. obovata*.

**Results:**

In this study, a *WRKY* transcription factor gene, named *KoWRKY40*, was identified from mangrove plant *K. obovata*. The full-length cDNA of *KoWRKY40* gene was 1420 nucleotide bases, which encoded 318 amino acids. The KoWRKY40 protein contained a typical WRKY domain and a C2H2 zinc-finger motif, which were common signatures to group II of WRKY family. The three-dimensional (3D) model of KoWRKY40 was formed by one α-helix and five β-strands. Evolutionary analysis revealed that KoWRKY40 has the closest homology with a WRKY protein from another mangrove plant *Bruguiera gymnorhiza*. The KoWRKY40 protein was verified to be exclusively located in nucleus of tobacco epidermis cells. Gene expression analysis demonstrated that *KoWRKY40* was induced highly in the roots and leaves, but lowly in stems in *K. obovata* under cold stress. Overexpression of *KoWRKY40* in *Arabidopsis* significantly enhanced the fresh weight, root length, and lateral root number of the transgenic lines under cold stress. *KoWRKY40* transgenic *Arabidopsis* exhibited higher proline content, SOD, POD, and CAT activities, and lower MDA content, and H_2_O_2_ content than wild-type *Arabidopsis* under cold stress condition. Cold stress affected the expression of genes related to proline biosynthesis, antioxidant system, and the ICE-CBF-COR signaling pathway, including *AtP5CS1*, *AtPRODH1*, *AtMnSOD*, *AtPOD*, *AtCAT1*, *AtCBF1*, *AtCBF2*, *AtICE1*, *AtCOR47* in *KoWRKY40* transgenic *Arabidopsis* plants.

**Conclusion:**

These results demonstrated that *KoWRKY40* conferred cold tolerance in transgenic *Arabidopsis* by regulating plant growth, osmotic balance, the antioxidant system, and ICE-CBF-COR signaling pathway. The study indicates that *KoWRKY40* is an important regulator involved in the cold stress response in plants.

**Supplementary Information:**

The online version contains supplementary material available at 10.1186/s12870-022-03661-2.

## Background

Mangrove plants are tropical/subtropical communities of xylophyta that grow in the intertidal zones of coastlines [1]. As an important marine wetland ecosystem, mangrove plants play important ecological and economic roles, such as keeping coastlines and beaches away from storm tide and tsunami, remediating contaminated environment, and providing food and shelters for marine organisms [2, 3]. As a dominant community, mangrove plants have evolved to adapt and survive in such extreme habitat (high ultraviolet radiation, high salinity, submerged and hypoxia soil) [4]. Generally, plants have developed complex mechanisms to adapt to stress conditions. Among them, some important transcription factors, such as WRKY transcription factor (TF) family, have participated in the response to various tresses by activating or repressing the expression of the related genes [5]. Since mangrove plants are typically materials for investigating plant adaptive traits [6], the WRKY TF genes might provide clues to understand the adaptation and stress resistance mechanisms of mangrove plants. However, the roles of the *WRKY* genes in mangrove plants responding to stress are still poorly understood.

The WRKY TFs, one of the largest transcription factor families, involved in various biological processes in plants [7]. WRKY family members have a highly conserved 60 amino acids of DNA-binding regions, called WRKY domain. The WRKY domain contains the conserved WRKYGQK sequence at N-terminal and zinc-finger structure at C-terminal [8]. The DNA binding domain of WRKY is mostly invariant WRKYGQK, but it still has differences from other domain, such as WKKYGQK, WRKYGMK, WSKYGQK, WKRYGQK, WVKYGQK, and WRKYGKK [9–11]. Almost all the known WRKY proteins with WRKYGQK sequence can recognize the W-box (TTGACC/T sequences) in promoter region [12]. The zinc-finger structures mainly contain C2H2 type (C-X_4–5_-C-X_22–23_-H-X_1_-H) and C2HC type (C-X_7_-C-X_23_-H-X_1_-C) [13]. Based on the number of WRKY domains and the structural feature of zinc-finger motifs, WRKY proteins can be divided into three groups, namely I, II, and III [13]. Group I WRKY proteins have two WRKY domains and a C2H2 zinc-finger structure. Group II WRKY proteins contain only one WRKY domain and a C2H2 zinc-finger structure. Finally, group III WRKY proteins have one WRKY domain and a C2HC-type zinc finger [8, 13, 14].

Many reports have suggested that WRKY proteins were involved in plant developmental processes and various abiotic defense responses. Studies showed that expression of *WRKY* genes were induced under cold and salt stresses in plants [15–18]. Additionally, overexpression of some *WRKY* genes had regulated the expression levels of many other stress-related genes, and also enhanced tolerance to salt, cold, heat, or drought stress in transgenic plants [18–21]. However, there was little study about *WRKY* genes in mangrove plants [6].

*Kandelia obovata* is widely distributed in the world and is the most cold-resistance species in mangrove plants [22]. In our previous study, the nucleotide fragment (Ko1140) isolated from *K. obovata*, and showed homology with other WRKY proteins [23]. In order to better understand it, the full-length sequence of this novel gene, named *KoWRKY40,* has been cloned and analyzed for its structure and function in this study. The evolutionary relationship of KoWRKY40 protein with other WRKY proteins was analyzed. The expression patterns of *KoWRKY40* in response to cold stress in different tissues of *K. obovata* were characterized. The subcellular localization of *KoWRKY40* was performed based on the expression of GFP fusion proteins in tobacco. Furthermore, overexpression of *KoWRKY40* in *Arabidopsis thaliana* was carried out for cold resistance analysis. This study provided useful clues for further exploring the functional mechanism of *KoWRKY40* in *K. obovata*.

## Results

### Characterization and sequence analysis of the *KoWRKY40*

**Fig. 1:**
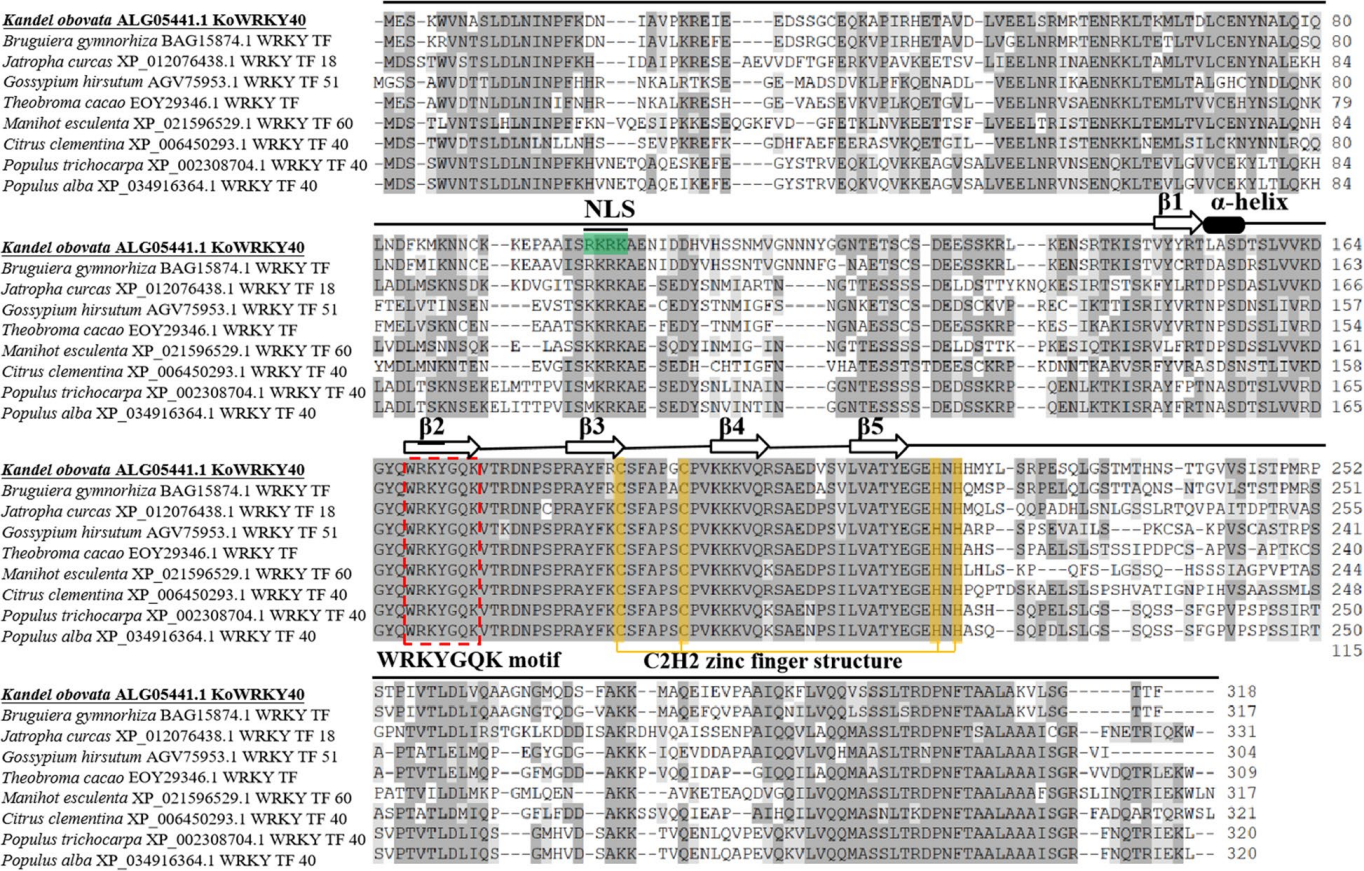
Sequence alignment of KoWRKY40 protein and other eight WRKY TFs from group II. The information of these protein sequence are as follows: ALG05441.1 KoWRKY40 of *Kandelia obovata*, BAG15874.1 WRKY TF of *Bruguiera gymnorhiza*, XP_012076438.1 WRKY TF 18 of *Jatropha curcas*, AGV75953.1 WRKY TF 51 of *Gossypium hirsutum*, EOY29346.1 WRKY TF of *Theobroma cacao*, XP_021596529.1 WRKY TF 60 of *Manihot esculenta*, XP_006450293.1 WRKY TF 40 of *Citrus clementina*, XP_002308704.1 WRKY TF 40 of *Populus trichocarpa*, XP_034916364.1 WRKY TF 40 of *Populus alba*. The similar and selfsame amino acids among WRKY proteins are marked light and dark gray, respectively. The predicted *α*-helix and five *β*-strands are indicated at the top of the sequence by a black box and five wide white arrows, respectively. Conserved WRKYGQK of WRKY proteins used to stabilize the structure and recognize DNA has been marked with a red dashed box. The yellow background indicates the conserved zinc finger motif (C2H2, C-X4–5-C-X22–23-H-X1-H). The residue of “RKRK”, highlighted in green, is the nuclear localization signal (NLS) of KoWRKY40

Initially, a fragment with 334 bp was obtained from our previous study, which has the homology with those known WRKY genes. According to RACE technology, the full-length cDNA of this WRKY gene was obtained by sequence assembly and re-amplification. Sequence analysis revealed that the cDNA fragment is 1420 bp in length, containing a 127-bp 5′-untranslated region (UTR), a 336-bp 3′-UTR and a 957-bp complete open reading frame (ORF). The gene encoded a protein with 318 amino acid residues with an estimated molecular mass (MW) of 33.59 kDa and isoelectric point (p*I*) of 8.76. This gene has been deposited in GenBank (GenBank accession No. KP267757.1) and was designated as *KoWRKY40* in this study. According to EXPASy Molecular Biology Server, the Ser (10.1%), Lys (7.9%), Thr (7.2%), Val (7.2%), Ala (6.9%), Asn (6.9%) Glu (6.9%) and Leu (6.6%) contents were high, but the Trp occupied the lowest (0.6%) portion in KoWRKY40 amino acid sequence. In the secondary structure of KoWRKY40, α-helix accounted for 41.19%, β-sheet for 16.35%, β-turn for 5.97%, and random coil for 36.48%. Bioinformatics analysis showed that KoWRKY40 contained two transmembrane regions (residue positions 45–60 and 299–314), but had no predicted signal peptide. A putative nuclear localization signal (NLS) RKRK is existed on the residue positions 99–102 (Fig. 1), showing that KoWRKY40 is located in the nucleus. The sequence alignment indicated that KoWRKY40 protein had high homology comparing with other eight WRKY TFs (Fig. 1). These nine WRKY TFs all contain a WRKYGQK sequence and a C2H2 zinc finger structure, which are the typical features of Group II WRKY proteins, indicating that they belong to the Group II WRKY subfamily (Fig. 1).

### Three-dimensional model of KoWRKY40

**Fig. 2:**
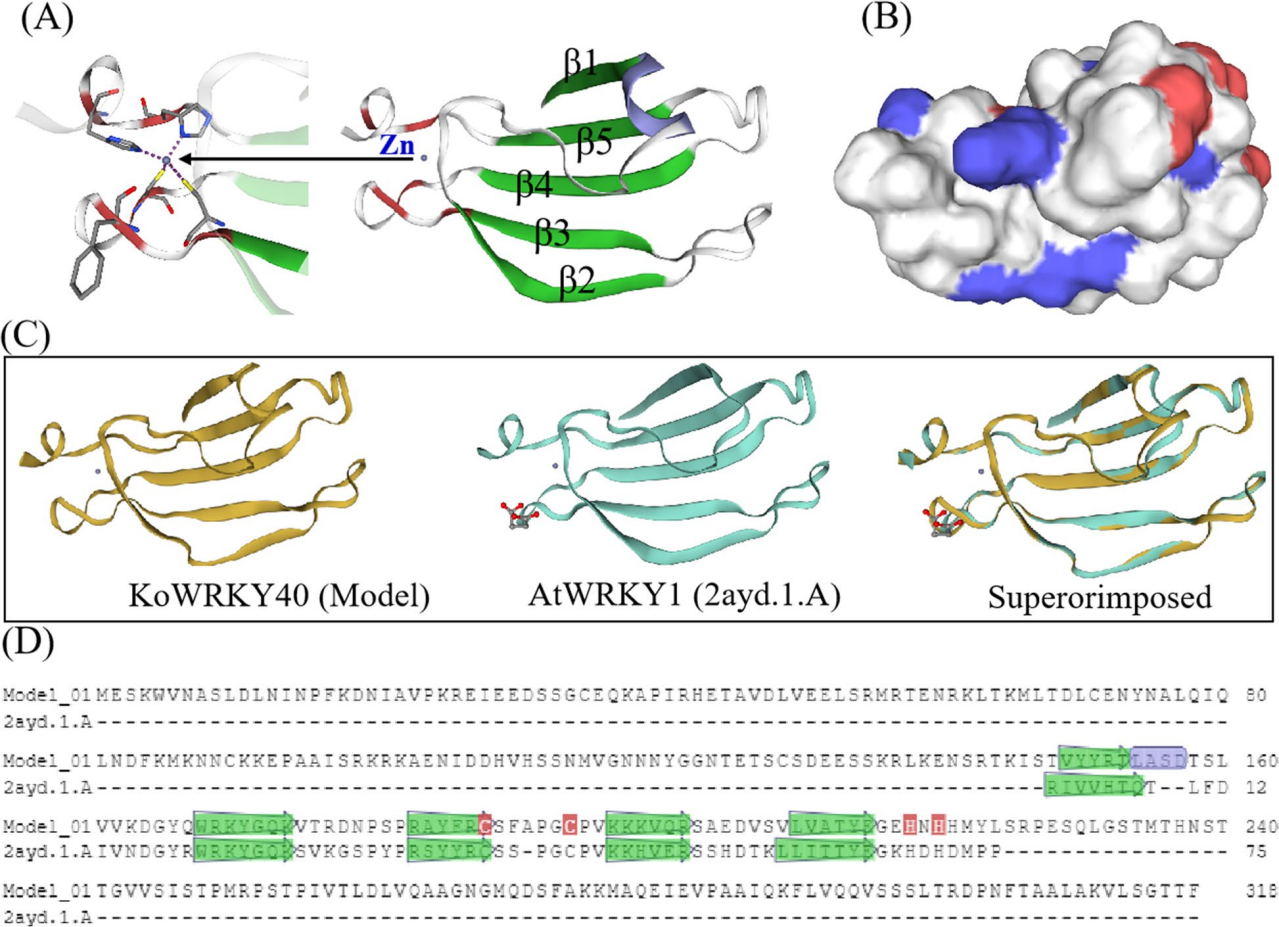
Structure of KoWRKY40 protein and comparison with its template AtWRKY1. The 3D structural model of KoWRKY40 was generated based on its template AtWRKY1 (SMTL id: 2ayd.1.A) through homology modeling SWISS-MODEL. The sequence identity between them is 51.35%. **A** Cartoon representation of KoWRKY40 protein. The KoWRKY40 is composed of one α-helix (purple curl) and five β-strands (green ribbons), which are numbered from the N-terminus. The claret regions of the cartoon structure represented zinc-binding sites. The zinc ion and zinc coordination residues are represented by purple spheres and rods (gray for C, red for O, blue for N, orange for S), respectively. **B** The charge distribution on the surface of KoWRKY40 structure. Negative charges are marked by red, and positive charges are indicated by blue. **C** 3D-superimposition structure of KoWRKY40 and the best representative structure of 2ayd.1.A. The macromolecular structures are shown by traces, with the KoWRKY40 colored in khaki and 2ayd.1.A in cyan. The zinc ions are shown by purple spheres in both KoWRKY40 and 2ayd.1.A. Succinic acid are represented by sticks in 2ayd.1.A. **D** Model-template alignment of KoWRKY40 and 2ayd.1.A. The β-strands were colored green, α-helix was colored purple. The claret residues represented zinc-binding sites, marked by yellow background in Fig. 1, which interacted with zinc-coordinating residues

Based on the deduced amino acid sequence, the predicted three-dimensional (3D) model of KoWRKY40 was constructed using SWISS MODEL software (Fig. 2). There is a 50% similarity between the amino acid sequences that need for the formation of the 3D model [24]. Modeling results showed that the sequence similarity was 51.35% between the KoWRKY40 and the template (AtWRKY1, SNYL id: 2ayd.1.A) [25], demonstrating the 3D model of KoWRKY40 was reasonable and credible. According to the surface charge distribution of 3D structure, positive charges occupied more area than negative charges in KoWRKY40 protein (Fig. 2B), indicating KoWRKY40 was a positive charge protein. Sequence analysis indicated that KoWRKY40 contained 38 negatively charged residues (Glu and Asp) and 43 positively charged residues (Lys and Arg), which means that KoWRKY40 was positively charged. In general, a positive charge indicates the hydrophobicity of the protein. Sequence analysis reconfirmed that the protein is indeed hydrophobic. The structure of KoWRKY40 was consisted of a five-strand anti-parallel β-sheet (β1, 149–153; β2, 168–174; β3, 183–188; β4, 197–202; β5, 210–215. see Fig. 2A). The 147 residues at the N-terminus (including the NLS motif) and the 94 residues at the C-terminus are not included in the structure (see Fig. 2D). Due to the α-helix (Leu154-Asp157) and the long bridging loop (Thr158-Gln167) between β1 and β2, the structure of KoWRKY40 looked spherical and stable (Fig. 2A, B). The zinc ion existed as an independent ligand and zinc coordination residues interact with amino acid residues at Cys188, Cys199, His218, His220, which are the core residues of the C2H2 zinc finger structure of KoWRKY40 (Fig. 2A). The C2H2 structure was located at one end of the β sheet (β5), between strands β3 and β4. The presence of zinc ion was essential for the DNA-binding activity, indicating the importance of the zinc-binding motif [14, 26]. In Fig. 1, there were more than 17 well-conserved residues in the area between β2 and β3, including WRKYGQK sequence. Since the WRKY motif is responsible for binding to W-box [14], and the ‘WRKYGQK’ sequence of WRKY motif spanned the entire β2 strand, indicating the importance of β2 of KoWRKY40 in the DNA-binding activity. Therefore, we infer that the β2 and β3 strands are likely to participate in DNA binding, and the loop between β2 and β3 may participate in conformational changes of DNA binding [25].

### Phylogenic analysis of KoWRKY40

**Fig. 3:**
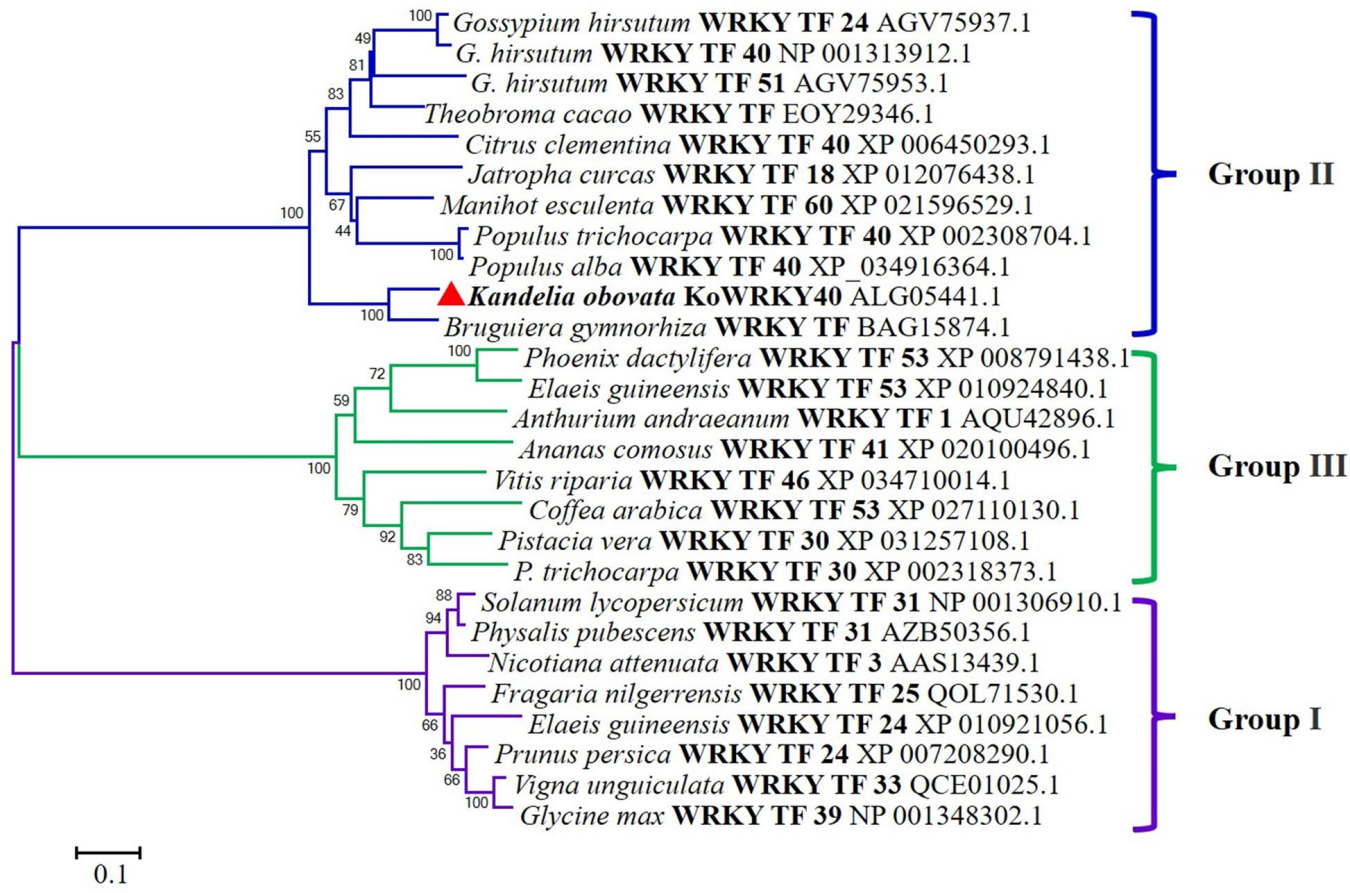
The phylogenetic analysis of KoWRKY40 protein. The phylogenetic tree consisted of 27 amino acid sequences from the NCBI database and marked with accession numbers. The red triangle represented KoWRKY40 protein. The scale indicates the length of the branch

We have downloaded all available *Arabidopsis* WRKYs by NCBI blast. The phylogenetic analysis of KoWRKY40 protein with *Arabidopsis* WRKYs was performed as shown in Supplementary Fig. S1. In comparing with the 58 *Arabidopsis* WRKYs, KoWRKY40 showed the closest phylogenetic relationship to WRKY transcription factor 40 of *A. thaliana* that belongs to Group II WRKY family. In order to further investigate the evolutionary relationship of KoWRKY40, 26 WRKY TF proteins that showed close homology with KoWRKY40 sequence by NCBI blast were downloaded and a phylogenetic tree was constructed (Fig. 3). These representative WRKY TFs were classified into three groups, and the clusters and groups were well-supported by the bootstrap values. The phylogenetic tree indicated that KoWRKY40 was more closely related to the Group II of WRKY family. Besides, the KoWRKY40 protein showed the closest phylogenetic relationship to BgWRKY, which was a putative WRKY protein from mangrove plant *Bruguiera gymnorhiza* (GenBank accession No. BAG15874.1) [27].

### Nuclear localization of KoWRKY40 in transiently transformed tobacco

**Fig. 4:**
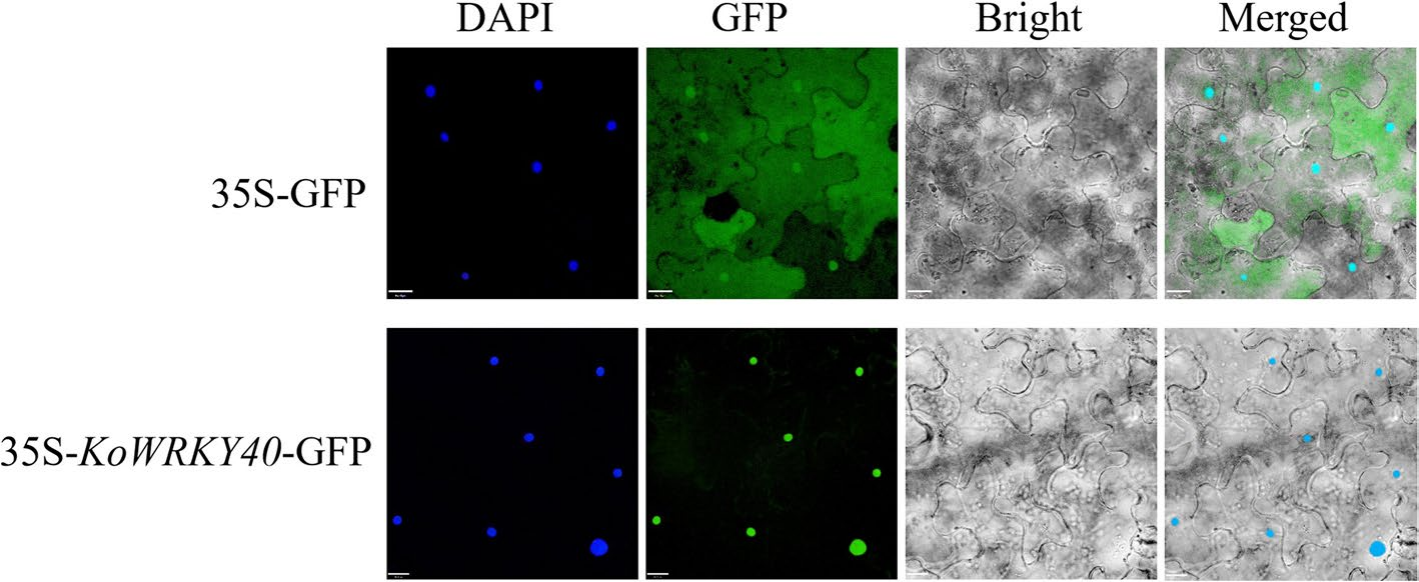
Subcellular localization of KoWRKY40. The fusion protein KoWRKY40-GFP (pCAMBIA2301-35S-KoWRKY40-GFP) and GFP (pCAMBIA2301-35S-GFP) were transiently expressed in *N. benthamiana* epidermal cells. Photographs were taken in bright and fluorescence fields after DAPI staining. Scale bar = 16 μm

As a transcription factor, WRKY proteins always possess NLS motif and were located in nucleus [28]. In order to verify the subcellular localization of KoWRKY40, the control vector 35S-GFP and the fusion expression vector 35S-*KoWRKY40*-GFP were transiently transformed into the epidermal leaf cells of tobacco *Nicotiana benthamiana* (Fig. 4), respectively. DAPI fluorescent dye was used as a nuclear marker. In leaves transformed with 35S-GFP vector, the green fluorescent signal of GFP was distributed through the cell. However, the green fluorescent signal was targeted specifically to the nucleus in leaves transformed with 35S-KoWRKY40-GFP (Fig. 4). These results demonstrated that KoWRKY40 is a nuclear-localized protein, and provided direct evidence for the nuclear localization of KoWRKY40.

### Expression patterns of *KoWRKY40* in response to cold stress

**Fig. 5:**
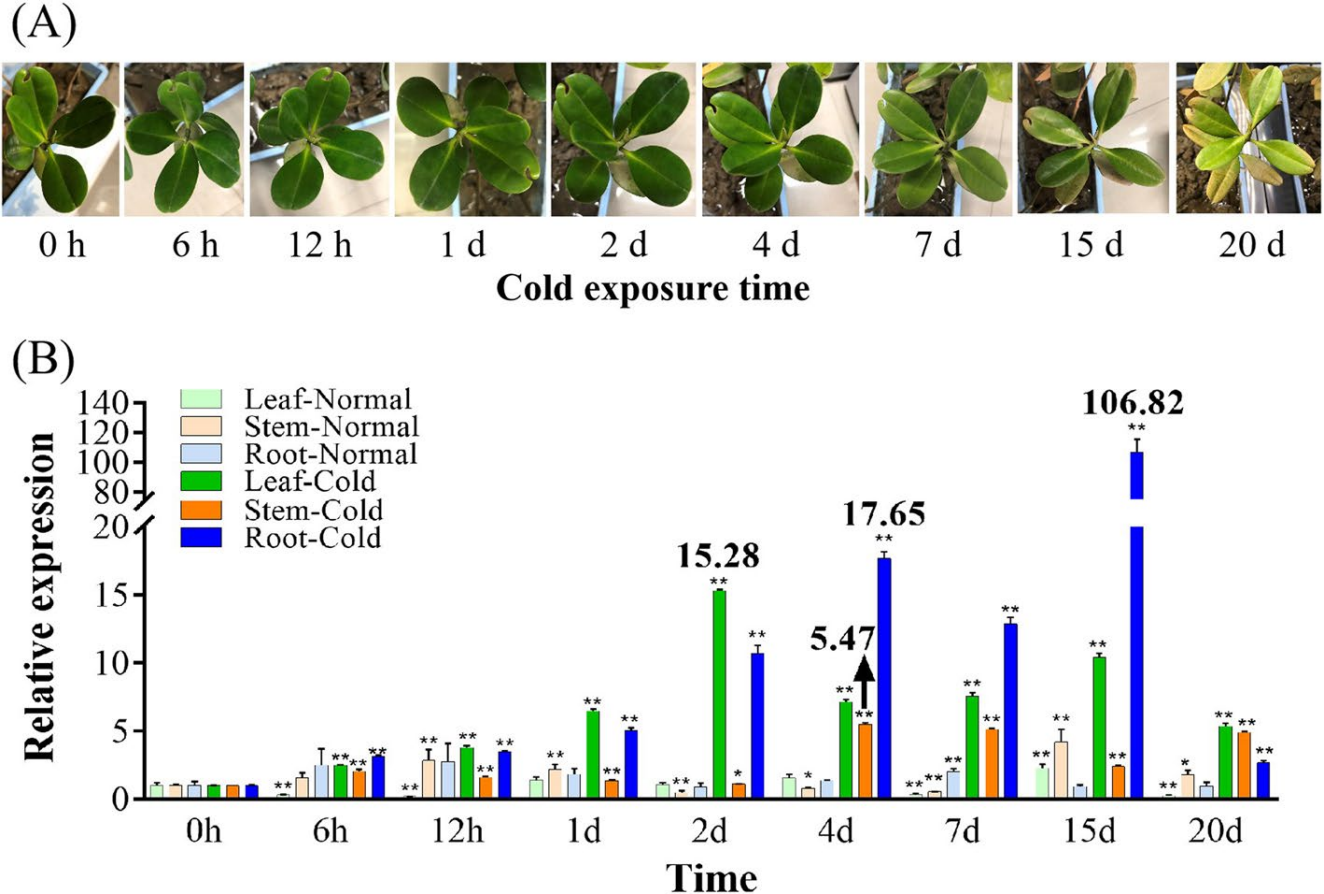
Morphological characters and expression of *KoWRKY40* of *K. obovata* under cold stress. **A** The morphological characters of *K. obovata* at different times under cold stress. **B** The relative expression levels of *KoWRKY40* in leaves, stems and roots under cold stress and normal condition. The relative expression of *KoWRKY40* were standardized using the expression of reference gene *Ko18S*. The data represented the average values of three biological repetitions. The error bars showed the standard deviations (*p* values was calculated by Student’s t test. *p < 0.05; **p < 0.01)

Cold stress usually affects plant growth. The leaves of *K. obovata* gradually withered with time extension under cold stress (Fig. 5A). Compared with normal condition (CK), the expressions of *KoWRKY40* were obviously induced in different tissues under cold stress (Fig. 5B). In leaves, the expressions of *KoWRKY40* were significantly higher under cold stress than that under normal condition during the whole tested-period (p* < 0.05). In stems and roots, the expressions of *KoWRKY40* were significantly increased under cold stress in comparison with normal condition (p* < 0.05) at 4 d, 7 d, 15 d and 20 d. In addition, the gene expressions of *KoWRKY40* were increased highly in the roots and leaves, but lowly in the stems under cold stress (Fig. 5B). During the tested time period, the expressions of *KoWRKY40* were all increased at first, and then decreased in leaves, stems and roots, respectively in cold-treatments. The difference was that the time points of the highest expression levels were different, at 2 d (15.28-fold), 4 d (5.47-fold) and 15 d (106.82-fold) in leaves, stems and roots, respectively. These data indicated that leaves were the first to respond to cold stress, followed by stems and roots. After 4-days cold treatment, the expression levels of *KoWRKY40* were higher in roots (17.65-fold) than that in leaves and stems under cold stress. These results suggested that *KoWRKY40* mainly reacted in the leaves at early phases (before 2 d), and principally played roles in roots at late phases (after 4 d) under cold stress. After 20-days cold treatment, the expressions of *KoWRKY40* in different tissues were decreased to low levels, probably because the plants withered at this time point (Fig. 5A). These results may suggest that the 20-days of cold treatment may have exceeded cold tolerance limit time of *K. obovata.* It was indicated that *KoWRKY40* may play important roles in the signaling network of *K. obovata* in response to cold stress.

### Overexpression of *KoWRKY40* enhanced the tolerance to cold stress in transgenic *Arabidopsis*

**Fig. 6:**
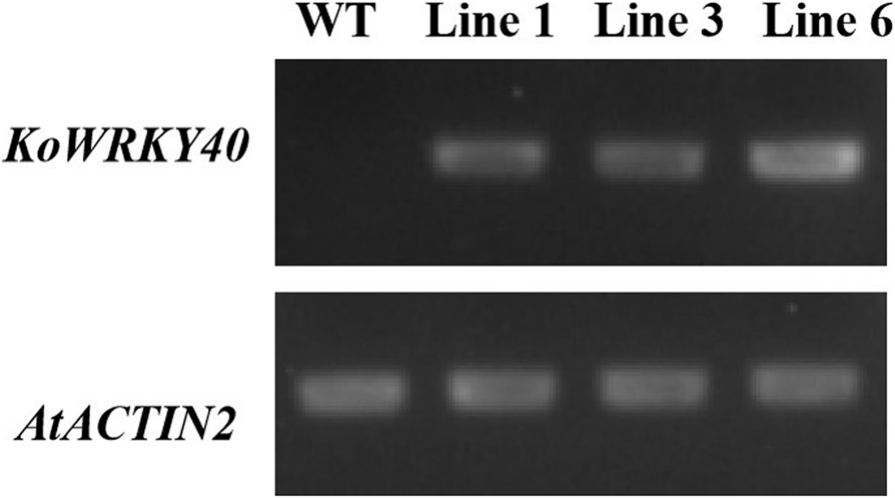
RT-qPCR identification of *KoWRKY40* expression in WT and *KoWRKY40* transgenic lines. The *AtACTIN2* gene was amplified as a control. *Lane* 1, WT, *lanes* 2–4, Line1, Line 3 and Line 6. Original gel was presented in Supplementary Fig. S2

**Fig. 7:**
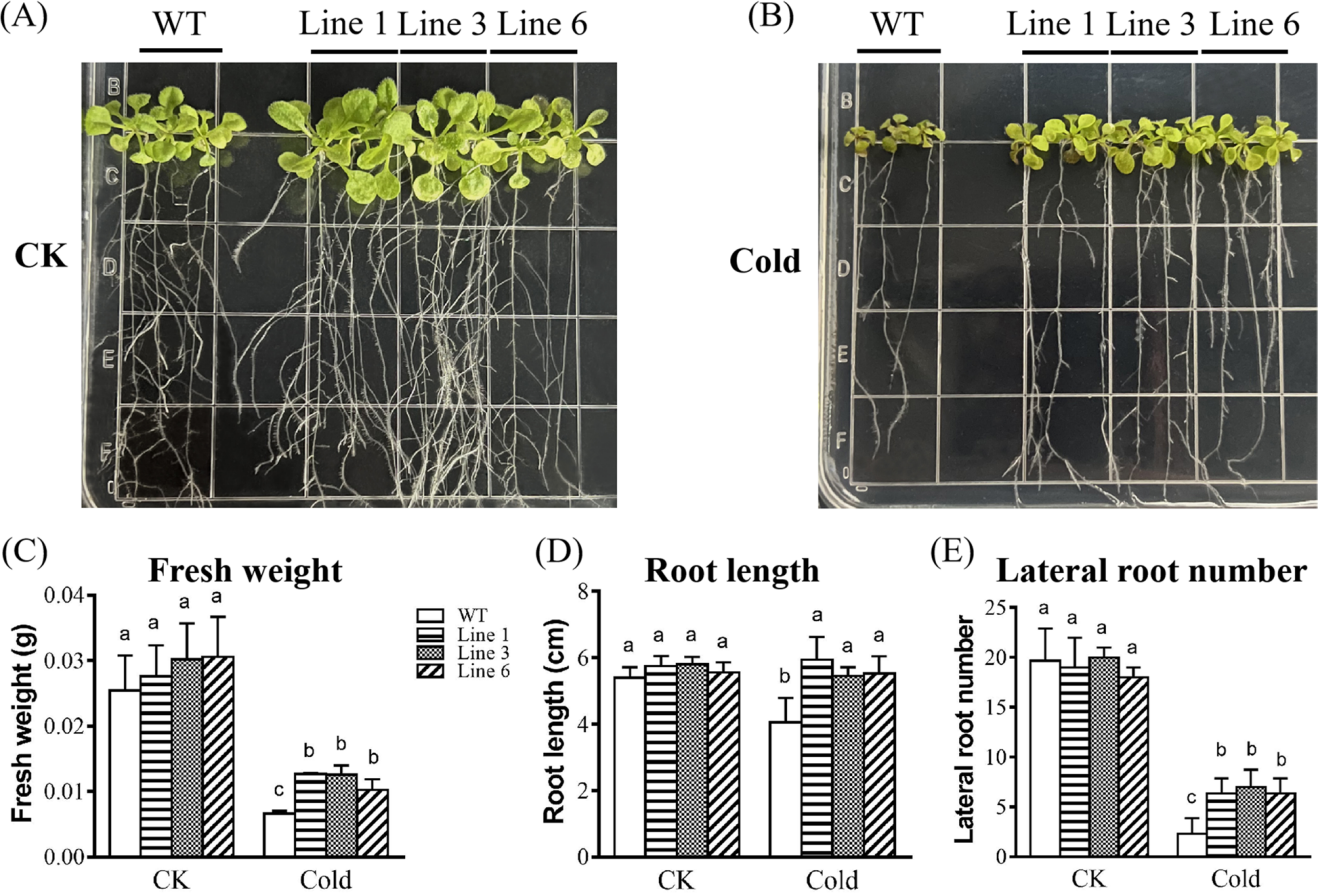
Phenotypic assay of *KoWRKY40* transgenic *Arabidopsis* under cold stress. Fifteen-day-old seedlings in MS agar medium under normal growth condition (**A**) and cold stress condition (**B**) for 10 days, respectively. Measurement of fresh weight (**C**), root length (**D**) and lateral root number (**E**) of *KoWRKY40* transgenic lines and wild-type plants. Line 1, Line 3 and Line 6 denote *KoWRKY40* transgenic lines 1, 3 and 6, respectively. WT denotes the wild type. Data were analyzed by one-way analysis of variance followed by Duncan’s test. Error bars with different letters represent statistically significant differences (*P* < 0.05, Duncan’s test)

To investigate the role of *KoWRKY40* in the response to cold stress, transgenic *Arabidopsis* plants that overexpressed *KoWRKY40* were generated. The wild-type (WT) *Arabidopsis* which has no expression of *KoWRKY40* transcript, and three *KoWRKY40* transgenic *Arabidopsis* lines (Line 1, Line 3 and Line 6) which demonstrated relatively high expression of *KoWRKY40* (Fig. 6), were selected and analyzed for stress tolerance. As shown in Fig. 7, both transgenic and wild-type (WT) seedlings grew well and showed no significant difference in phenotype, fresh weight, root length, and lateral root number under normal growth condition (CK). Nevertheless, the transgenic lines showed more green leaves, less black or yellow leaves than the wild type under cold stress (Fig. 7B). Besides, the fresh weight (Fig. 7C), root length (Fig. 7V), and lateral root number (Fig. 7E) of *KoWRKY40* transgenic *Arabidopsis* plants were significantly higher than that of WT plants under cold stress condition. The results showed that *KoWRKY40* transgenic lines grew better than WT plants under cold stress condition.

**Fig. 8:**
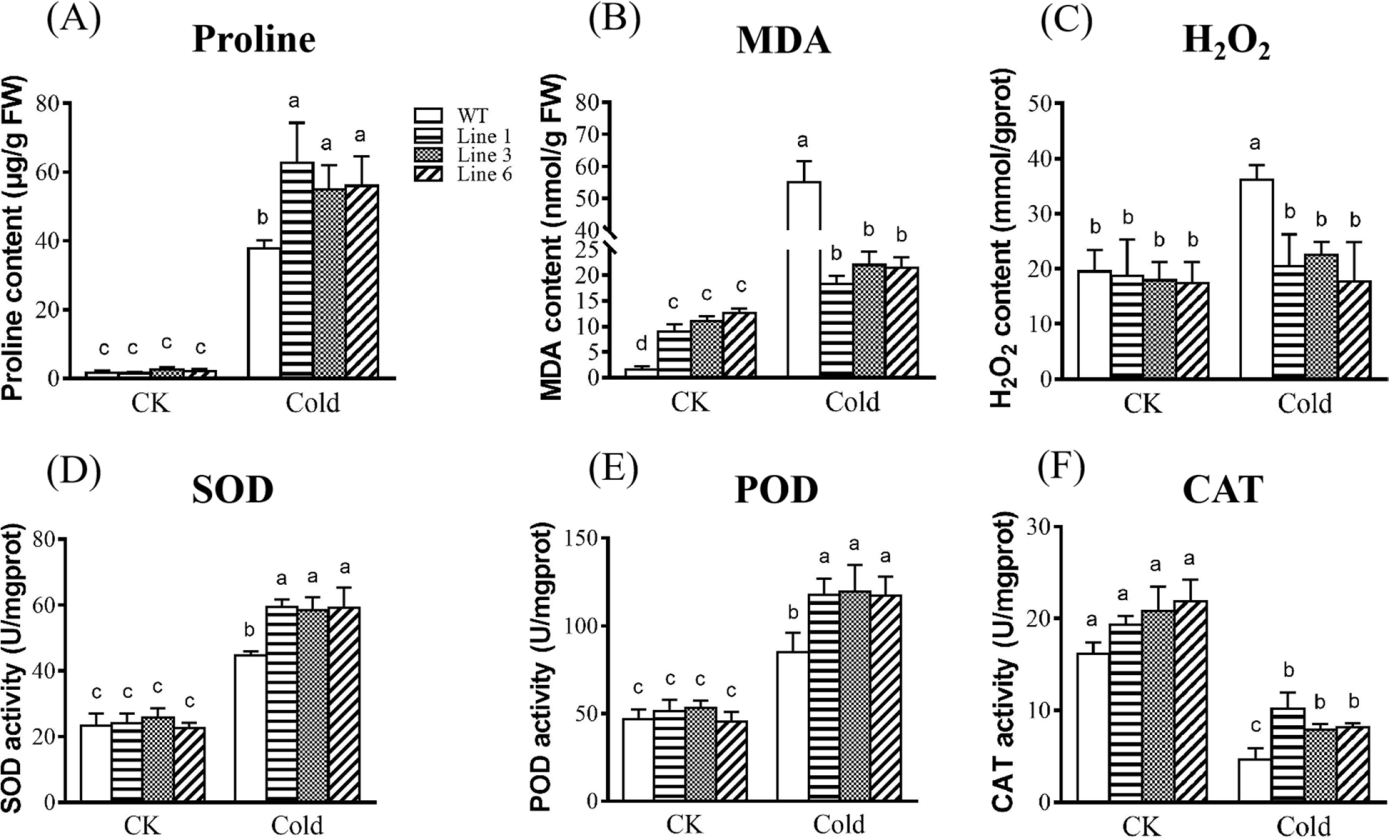
Measurement the contents of proline, MDA, and H_2_O_2_, and the activities of SOD, POD, and CAT in *KoWRKY40* transgenic lines and wild-type plants under cold stress. Line 1, Line 3 and Line 6 denote *KoWRKY40* transgenic lines 1, 3 and 6 respectively. WT denotes the wild type. Data were analyzed by one-way analysis of variance followed by Duncan’s test. Error bars with different letters represent statistically significant differences (*P* < 0.05, Duncan’s test)

To explore the involvement of *KoWRKY40* in osmoregulation in *Arabidopsis* under cold stress condition, the proline content was measured in WT and transgenic plants. As shown in Fig. 8A, the proline content was significantly higher in transgenic lines than in WT lines under cold stress condition, but did not differ significantly between the two types of plants under normal growth condition. These results suggested that overexpression of *KoWRKY40* enhanced the osmoregulatory capacity of *Arabidopsis* plants by increasing the proline content of plant cells, conferring cold tolerance to transgenic plants. To confirm the involvement of *KoWRKY40* in the antioxidant function of *Arabidopsis* under cold stress condition, the malondialdehyde (MDA) content, hydrogen peroxide (H_2_O_2_) content, and the activities of superoxide dismutase (SOD), peroxidase (POD), catalase (CAT), were examined in WT and *KoWRKY40* transgenic lines. As shown in Fig. 8B-F, MDA content and H_2_O_2_ content were significantly lower, whereas SOD, POD, and CAT activities were higher in *KoWRKY40* transgenic lines than in WT plants under cold stress condition. These results indicated that overexpression of *KoWRKY40* decreased membrane damage, ROS (reactive oxygen species) level, and enhanced the efficiency of antioxidant systems *Arabidopsis* under cold stress condition.

**Fig. 9:**
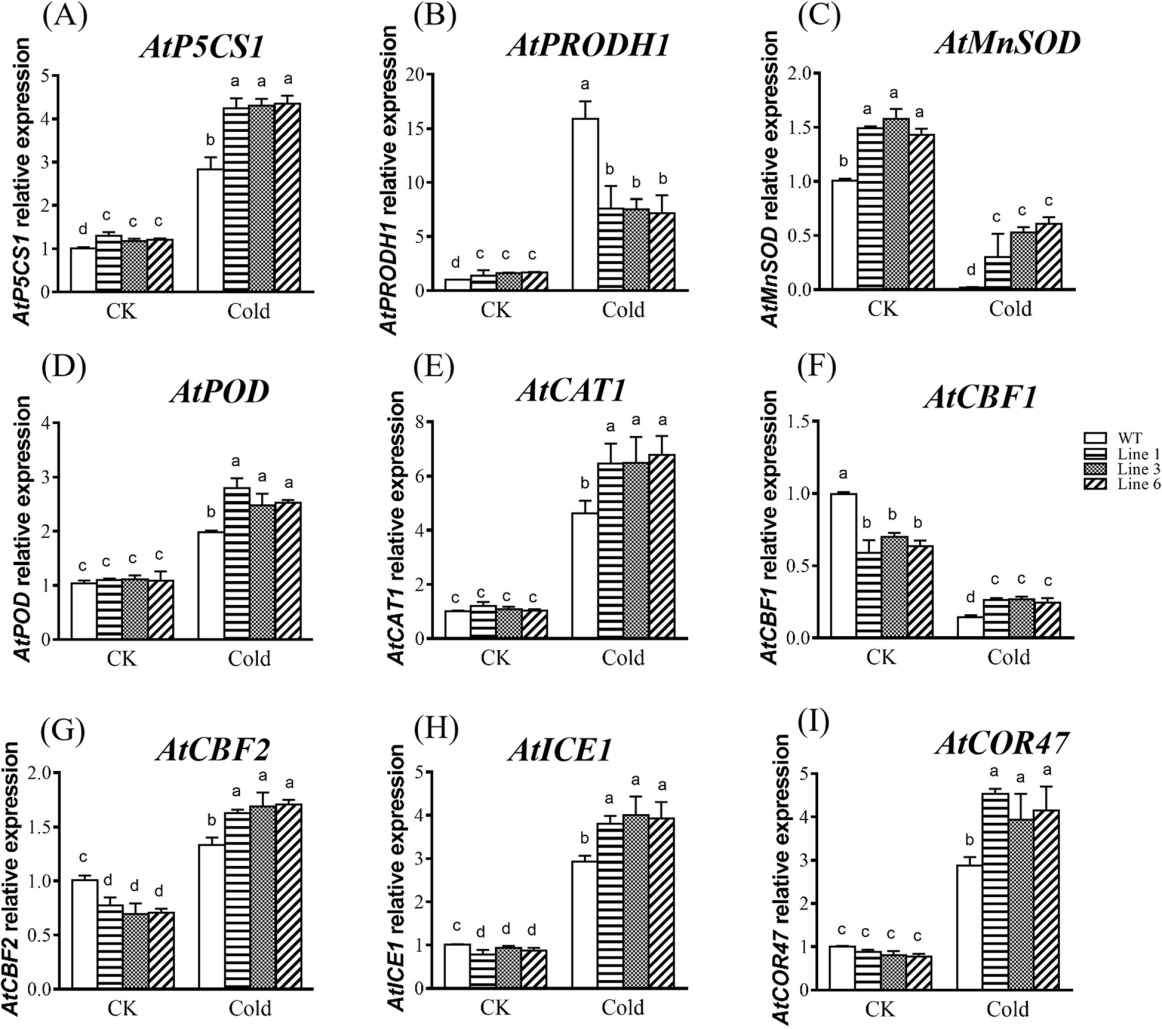
The expression of the stress-responsive genes *AtP5CS1*, *AtPRODH1*, *AtMnSOD*, *AtPOD*, *AtCAT1*, *AtCBF1*, *AtCBF2*, *AtICE1,* and *AtCOR47* in *KoWRKY40* transgenic lines and wild-type plants under cold stress. Relative expression indicates expression levels of tested genes in transgenic plants compared to their expression levels observed in WT plants under normal growth condition. All values were normalized to the Arabidopsis *AtACTIN2* expression level. Line 1, Line 2, and Line 3 denote *KoWRKY40* transgenic lines 1, 2, and 3, respectively. WT denotes the wild type. Data were analyzed by one-way analysis of variance followed by Duncan’s test. Error bars with different letters represent statistically significant differences (*P* < 0.05, Duncan’s test)

To further investigate the regulatory role of *KoWRKY40* in response to cold stress, the expression levels of important genes involved in osmotic adjustment (*AtP5CS1* and *AtPRODH1*), ROS scavenging (*AtMnSOD*, *AtPOD* and *AtCAT1*) and ICE-CBF-COR signaling pathway (*AtCBF1*, *AtCBF2*, *AtICE1* and *AtCOR47*) were examined in *KoWRKY40* transgenic and WT plants. The expression levels of *AtP5CS1*, *AtMnSOD*, *AtPOD*, *AtCAT1*, *AtCBF1*, *AtCBF2*, *AtICE1* and *AtCOR47* were significantly higher in *KoWRKY40* transgenic lines than in WT plants (as shown in Fig. 9). However, the expression level of proline dehydrogenase gene, *AtPRODH1*, was remarkably lower in *KoWRKY40* transgenic lines than in WT plants (Fig. 9B). These results suggested that overexpression of *KoWRKY40* in *Arabidopsis* regulated the expression of genes related to osmolytes, antioxidant biosynthesis, and ICE-CBF-COR signaling pathway under cold stress condition, generating transgenic *Arabidopsis* plants with improved osmoregulation, antioxidant defenses, and cold-related genes activation, thereby conferred to cold tolerance.

## Discussion

Many researches have reported that the WRKY proteins play important roles in response to abiotic stresses [29–31] Although there have been many studies on WRYK gene in other plants [8, 13, 14], little was known about WRKY genes in mangrove plants [6]. In this paper, based on our previous study [23], we cloned the full-length sequence of *KoWRKY40* gene from *K. obovata*. The deduced amino acid sequence showed that KoWRKY40 was a group II WRKY protein.

In general, WRKY proteins are characterized by their domains, which are typically about 60 residues in length and has a conserved WRKYGQK motif. The WRKYGQK motif is a W-box element (C/T) TGAC (C/T) located in the promoter region of the target genes and is essential for DNA binding activity [14]. The 3D structure of WRKY4 exactly showed that conserved WRKYGQK residues can participate in DNA binding [32]. However, the core WRKYGQK sequence of WRKY protein is sometimes replaced by WRKYGKK, which is common variant in canola [33], tomato [9], and pepper [34]. It has been found that each amino acid residue of WRKYGQK sequence was replaced, the binding affinity with DNA was significantly decreased [26]. In our study, the KoWRKY40 possessed the WRKYGQK motif. Therefore, we speculated that the KoWRKY40 may recognize the W-box based on its typical WRKYGQK motif.

The transmembrane helix is required for membrane-associated proteins related to cell signal transduction and substrate transport [35]. Sequence analysis showed that the KoWRKY40 protein contained two transmembrane helixes, indicating the KoWRKY40 might actively participate in signal transduction. Previous researches had reported that most WRKY proteins contained a basic NLS [36], and GFP-WRKY fusion protein have demonstrated that diverse WRKY TFs were located in nucleus [20, 36, 37]. In this study, KoWRKY40 protein contained the NLS sequence RKRK and was predicted to be localized in the nucleus. Some studies have reported that merged fluorescence signals of GFP and DAPI were visualized for subcellular localization [38, 39], the DAPI staining was used to detect the florescent signals of nuclei for further confirmed the nuclear localization of KoWRKY40. Subcellular localization analysis further confirmed that KoWRKY40 was nuclear-localized protein in this study. These data indicated that our results were consistent with previous studies.

Some studies have reported that the expression of WRKY was tissue specific under abiotic stresses. For example, the *LcWRKY5* gene was expressed only in leaves and roots in sheepgrass [40]. In *Arabidopsis*, the *AtWRKY25* gene was mainly expressed in roots [41]. The expression of *CsWRKY2* in leaves was higher than in other organs in defense response in tea plant [20]. In our study, the *KoWRKY40* gene was highly induced in the roots and leaves under cold stress in *K. obovata*. Thus, we speculated that *KoWRKY40* may play an important role in *K. obovata* under cold stress.

It was worth mentioning that the expression level of *KoWRKY40* was increased to the highest at 15 d (106.82-fold) in roots under cold stress, but that was dramatically decreased at 20 d (2.64-fold). This variation tendency of *KoWRKY40* expression levels was usually agreement with morphological changes. Seedlings that had been cold-treated (5 °C) for 15 days were still alive after being cultured for 20 d at the recovery temperature (25 °C). However, seedlings that had been cold-treated (5 °C) for 20 days have withered and fallen (data not shown here) under the same recovery condition. These results suggested that the expression of *KoWRKY40* may be closely related to the survival of *K. obovata*, and play important roles in protecting *K. obovata* from cold stress. Based on the high consistency between expression of *KoWRKY40* at the molecular level and recovery survival at morphological level, we speculated that 15–20 days may be the upper limit of survival time of *K. obovata* seedlings at low temperature (5 °C).

WRKY proteins have involved in the response of plants to various stresses, and their homologs have been found in various plant species [29]. Several WRKY proteins were found to be induced in response to cold stress in maize [42], cotton [43] and barley [44]. However, there was an evidence reported that one tobacco *WRKY* gene was specifically induced during collaborative pressure of drought and heat, but not by drought or heat stress alone, indicating this *WRKY* genes were induced only by a combination of different stresses [45]. In this study, the *KoWRKY40* expression was induced during the entire process under cold stress, indicating the *KoWRKY40* gene could be induced by cold stress individually. Since mangrove plants generally grow in the intertidal zones and were subjected to various stresses [1–3], whether other *WRKY* genes of mangrove plants were induced by individual or combined stresses remains to be studied.

WRKY protein can activate or inhibit the transcription of downstream by combining with W-box cis-acting elements of target genes [8, 46]. Since W-box elements existed in most WRKYs, the WRKYs can bind with their own promoters to achieve self-regulation or cross-regulation networks [47]. *CaWRKY6* transcriptionally activated *CaWRKY40*, and conferred the plant more tolerant to high temperature and humidity in pepper [48]. *AtWRKY34* had a negative regulatory effect on cold response pathway, inducing enhanced resistance to cold stress [49]. WRKY proteins could be quickly and differentially expressed, regulating the expression of downstream genes and promoting signal transduction [41]. For example, the antioxidant enzyme genes *AtSOD*, *AtCAT*, and *AtAPX1*, which can scavenge reactive oxygen species (ROS) to protect plants from oxidative damage [50], are important downstream genes regulated by WRKY TFs. In our study, the expression levels of *AtMnSOD*, *AtPOD*, and *AtCAT1* were up-regulated in *KoWRK40* transgenic *Arabidopsis* plants under cold stress, acceleration of which could increase tolerance to cold stress in transgenic *Arabidopsis* plants.

The plant response to cold stress is rather complex. A frequent plant response to abiotic stress is to accumulate certain osmolytes, particularly proline [51–53], which then function as osmoprotectants. In *Arabidopsis*, the genes encode P5CS isoforms involved in proline biosynthesis, *AtP5CS1* (AT2G39800), and the genes encode ProDH isoforms involved in the degradation of proline, *AtPRODH1* (AT3G30775) [54–56]. In this study, the proline biosynthesis gene *AtP5CS1* was up-regulated, and the proline degradation gene *AtPRODH1* was down-regulated in *KoWRKY40* transgenic *Arabidopsis*, compared with their expression in WT plants (Fig. 9E, F). This suggested that *KoWRKY40* increased the proline content to enhance the osmoregulatory capacity of transgenic *Arabidopsis* in response to cold stress by inducing proline biosynthesis genes and inhibiting proline degradation genes. Cold stress also can induce the rapid generation and accumulation of ROS, resulting in secondary oxidative stress in plants [57]. Antioxidant enzymes (e.g., SOD, POD and CAT) can scavenge ROS to protect plants from oxidative damage [50]. The increase of cold tolerance in plants may due to the high expression of these genes [58–60]. In the present study, *KoWRKY40* transgenic plants exhibited higher activities of SOD, CAT, POD, and lower contents of MDA and H_2_O_2_ than WT *Arabidopsis* under cold stress conditions (Fig. 8B-F). Three genes related to ROS scavenging, *AtMnSOD*, *AtPOD*, and *AtCAT1*, were up-regulated in *KoWRKY40* transgenic *Arabidopsis* (Fig. 9C-E). Thus, our data suggested that *KoWRKY40* increased the activities of antioxidant enzymes, and decreased membrane system damage in transgenic *Arabidopsis* plants under cold stress condition. In *A. thaliana*, *AtCBF1*, *AtCBF2*, *AtICE1* and *AtCOR47* genes involved in ICE-CBF-COR signaling pathway have been proved to play important roles in cold tolerance [61–65]. In our study, overexpression of *KoWRKY40* enhanced cold tolerance in transgenic *Arabidopsis* plants and these cold-resistance genes were all significantly increased in *KoWRKY40* transgenic *Arabidopsis* plants. Thus, we suggested that accumulation of which may help to develop cold acclimation and protect transgenic plants from cold damage.

## Conclusions

To summarize, *KoWRKY40*, a new WRKY transcription factor gene from mangrove plant *K. obovata* was reported in this study. The KoWRKY40 protein was a nuclear-localized protein and a member of group II WRKY family. The expression of *KoWRKY40* was highly induced in the roots and leaves in *K. obovata* under cold stress. In addition, *KoWRKY40* enhanced cold tolerance of transgenic *Arabidopsis* lines by activating different stress responsive genes involved in osmotic adjustment (*AtP5CS1* and *AtPRODH1*), ROS scavenging (*AtMnSOD*, *AtPOD* and *AtCAT1*) and ICE-CBF pathway (*AtCBF1*, *AtCBF2*, *AtICE1*, *AtCOR47*). These results provide key insight into the role of this gene in *K. obovata* that *KoWRKY40* acts as a positive regulator of cold stress tolerance. This study suggested that *KoWRKY40* may be a valuable genetic resource in molecular breeding programs of plants. Future studies are needed to elucidate the functional mechanism of *KoWRKY40* against abiotic stresses.

## Methods

### Plant material, growth conditions and treatments

The hypocotyls of *K. obovata* were provided by Guangdong Mangrove Ecological Technology Co. Ltd. (China). The hypocotyls were surface-disinfected and sown in clean sand at room temperature. The 3-months seedlings were transferred into growth chamber with normal condition (25 °C, relative humidity 75%, 14 h light/10 h dark cycle). After 7 days, the seedlings were treated under cold condition (5 °C) for 0 h, 6 h, 12 h, 1 d, 2 d, 4 d, 7 d, 15 d and 20 d. Seedlings treated at 5 °C for 0 h were used as the control. All treatments contained at least three seedlings. The harvested leaves, stems and roots were immediately frozen in liquid nitrogen, and then transferred to − 80 °C refrigerator until use.

### RNA extraction and reverse transcription

Total RNA was extracted from leaves, stems and roots of *K. obovata* using the Tiangen RNA plant Plus Reagent (Tiangen Biotech, Beijing) according to the method as described [66, 67]. The 1.0% agarose gel was used to analyze the integrity and purity of total RNA. The quality and content of RNA were determined by Nanodrop 1000 spectrophotometer (Thermo Scientific, USA). The RNase-free DNase I (Promega, USA) was used to remove potential genomic DNA contamination of total RNA. The RNA samples was then subjected to synthesize the first strand cDNA by SMART™ reverse transcription Kit (Clontech, USA) following the manufacturer’s protocol. The cDNA samples were used for cloning the full-length of *KoWRKY40* gene and RT-qPCR analysis.

### Cloning the complete sequence of *KoWRKY40* gene

The rapid amplification of cDNA ends (RACE) PCRs was performed from the first cDNA strand of *K. obovata* leaves. A complete cDNA sequence was assembled by combining the 5′-RACE and the 3′-RACE fragments. Based on the partial sequences (GenBank accession number: JZ585678.1) from our previous study [23], the gene-specific primers (GSP1, 5′-GCTACTAGGACTGAAACATCCTCCGCAC-3′, GSP2, 5′- GGTGGCAATACTGAAACCAGCTGTAGCGA-3′) were used as primary PCR to obtain 5′ and 3′ end sequences, respectively. The nested PCR reactions were performed by nested primers (NGSP1, 5′-ATCCTCCGCACTTCTCTGGACCTTCTTC-3′, NGSP2, 5′-GAAACCAGCTGTAGCGACGAAGAGTCAA-3′). All steps of RACE were carried out using SMART™ RACE cDNA Amplification Kit (Clontech, USA) on the basis of the manufacturer’s protocol. The RACE products were purified by agarose gel and then were cloned into pMD19-T Vector for sequencing. The 3′- and 5′- nucleotide sequences were assembled by DNAMAN software through overlap to acquire the full-length *KoWRKY40* sequence. The assembled sequence was used to design the primers, and the full-length sequence of *KoWRKY40* was cloned and sent to the company (BGI, China) for sequencing. Finally, the complete cDNA sequence of *KoWRKY40* was confirmed, and was submitted to GenBank to obtain the accession number KP267757.1.

### Bioinformatic analysis

The DNAMAN software was used to obtain full-length cDNA sequence through overlap fragments by linking sequences. NCBI BLAST tools (http://blast.ncbi.nlm.nih.gov/Blast.cgi) was applied for sequence analyses and comparisons. The deduced amino acid sequence of KoWRKY40 (GenBank accession number: KP267757.1) was inferred by ORF Finder (http://www.ncbi.nlm.nih.gov/gorf/gorf. html). Compute p*I*/MW tool (http://web.expasy.org/compute_pi/) was used to analyze the molecular mass and isoelectric point of *KoWRKY40*. The trans-membrane domain prediction was analyzed by TMpred (http://www.ch.embnet.org/software/TMPRED_form.html). The Motif Scan (http://myhits.isb-sib.ch/cgi-bin/motif_scan) was used to detect motif sequences. The subcellular localization was predicted by PSORT (http://www.psort.org/) and Softberry ProComp v. 9.0 (http://linux1.softberry.com/all.htm). The signal peptides of *KoWRKY40* were predicted using SignalP 4.1 Server (http://www.cbs.dtu.dk/services/SignalP/). Multiple alignments of the diverse WRKY proteins were generated by the BioEdit software. The phylogenetic tree of WRKY proteins was constructed by Clustal X software and MEGA 5.0 software with the neighbor-joining algorithm. The reliability of the phylogenetic tree was tested by bootstrap analysis with 1000 replicates. The SOPMA (https://npsa-prabi.ibcp.fr/cgi-bin/npsa_automat.pl?page=/NPSA/npsa_ sopma.html) was used to predict the secondary structure of the KoWRKY40. The tertiary structure of KoWRKY40 was predicted by Swiss-Model tool (http://www.swissmodel.expasy.org/).

### Expression analysis by RT-qPCR

To analyze *KoWRKY40* expression in *K. obovata* under cold stress, the real-time quantitative PCR (RT-qPCR) was performed to determine the transcription levels in leaves, stems and roots of *K. obovata*. The samples were collected from different tissues of *K. obovata* at 0 h, 6 h, 12 h, 1 d, 2 d, 4 d, 7 d, 15 d and 20 d, respectively, under cold condition (5 °C). For comparison, the samples were also collected at same time points under normal condition (25 °C). The samples collected at 0 h were considered as the control. Total RNA was extracted and reverse-transcribed into cDNA in the above experiment. The internal reference gene was selected with the *18S* (*Ko18S*) rRNA of *K. obovata*. The RT-qPCR reactions were carried out by iCycler iQ5 real time PCR detection system (Bio-Rad, CA, USA) according to SYBR Premix Ex TaqTM II (Takara, Dalian, China) manufacturer’s instructions. The RT-qPCR reaction parameters were 95 °C for 1 min, 40 cycles at 95 °C for 10 s, 55 °C for 30 s, and 72 °C for 40 s. The 2^-△△CT^ method was used to calculate the relative expression levels of gene [68, 69] comparing with 0 h. Sequences of primers used for RT-qPCR analysis are listed in Supplementary Table S1. Three biological replicates and three technical replicates were performed for each sample. The data was expressed as mean ± standard deviation (x ± SD). Statistical analyses were carried out by Student t-test, and diagramming was performed with GraphPad Prism 7.0 (GraphPad Software, San Diego, California). One-way ANOVA was selected to quantify the significant differences among expressions of *KoWRKY* under cold stress and normal condition by SPSS statistics 25.

### Subcellular localization analysis

To determine the subcellular localization of KoWRKY40, the ORF of *KoWRKY40* termination-codon free was cloned into vector pCAMBIA2301-35S-GFP. The *Nicotiana benthamiana* leaf preparation and transformation were performed as described [39]. The GFP fusion expression vector pCAMBIA2301-35S-*KoWRKY40*- GFP was analyzed and sequenced, and the successful fusion was confirmed. The recombinant plasmid was introduced into *Agrobacterium tumefaciens* strain EHA105, and transformed transiently into the leaf epidermal of *N. benthamiana* by *Agrobacterium* infection. The 35S-GFP vector was taken as the control. To locate the fluorescent proteins in nuclei, the *N. benthamiana* leaves were infiltrated with PBS containing 4′,6-diamidino-2-phenylindole (DAPI) for 20 min, and the fluorescence microscopic image was observed by Zeiss LSM710 laser scanning confocal microscope to determine the subcellular location of KoWRKY40-GFP.

### Generation of *KoWRKY40* transgenic *Arabidopsis* plants

For *KoWRKY40* overexpression in wild-type *Arabidopsis thaliana* (WT; Columbia), the full-length *KoWRKY40* sequence without the stop codon was cloned into pCAMBIA2301 and driven by CaMV 35S promoter. The coding sequence of *KoWRKY40* (with KpnIsite added to its 5′ and 3′ ends, respectively) was amplified from pMD19-T-*KoWRKY40* using gene-specific primers F1 (5′-GCGGGTCGACGGTACCATGGAATCAAAATGGGTGAAC-3′) and R1 (5′-TAGACATATGGGTACCGAATGTGGTTCCTGAAAGG-3′). The digested amplicon was inserted into pCAMBIA1301 driven by the CaMV 35S promoter, and confirmed by sequencing. This recombinant vector, named *pCAMBIA2301*-*35S::KoWRKY40*, was transformed into *A. tumefaciens* EHA105 by the freeze-thaw method and then transformed into *Arabidopsis* plants by the floral dip method [70]. Positive *Arabidopsis* transgenic lines were harvested and selected by culturing on MS medium agar plates containing 50 mg/L kanamycin, and these were advanced by self-pollinated until obtaining T_3_ transgenic plants. Transgenic plants were validated further by RT-PCR (reverse transcription-PCR) analysis with gene-specific primers F2 (5′-GACGCACAATCCCACTATCC-3′) and R2 (5′-GAATGTGGTTCCTGAAAGG-3′). Finally, the T3 or T4 homozygous lines were used for all the subsequent experiments.

### Physiological analysis of transgenic *A. thaliana* lines under cold stress

Seeds of WT *Arabidopsis* and *KoWRKY40* transgenic *Arabidopsis* lines (Lines 1, 3 and 6) were surface sterilized by soaking in 70% ethanol (v/v) for 5 min, and then rinsed four to five times with sterile distilled water. The sterilized seeds were grown on solidified MS medium for 6 days and transplanted onto new square plates with MS medium under normal conditions for 10 days before treatments. For cold tolerance evaluation, some wild-type and *KoWRKY40* transgenic plants were cultured under normal conditions as the control and others were treated under cold stress (5 °C) for 10 days, and their fresh weight, root length, lateral root number and other physiological parameters were measured. The contents of proline, MDA, and H_2_O_2_, and the activities of SOD, POD, and CAT, were measured using the corresponding assay kits (Jiancheng Bioengineering Institute, Nanjing, China) according to the manufacturer’s instructions. The data was expressed as mean ± standard deviation (x ± SD). All the experiments were carried out in triplicate for biological replicates. Statistical analysis was performed with GraphPad Prism 7.0 (GraphPad Software, San Diego, California). One-way ANOVA followed by Duncan’s test was selected to quantify the significant differences by SPSS statistics 25.

### Expression analysis of stress-related genes in transgenic *A. thaliana* lines

To examine the expression of stress-related genes, cDNA was synthesized from RNA extracted from the leaves of WT Arabidopsis and *KoWRKY40* transgenic lines (Lines 1, 3 and 6). The expression levels of cold-related genes were measured by qRT-PCR as described above. The stress-related genes monitored were *AtP5CS1* (AT2G39800), *AtPRODH1* (AT3G30775), *AtMnSOD* (AT3G56350), *AtPOD* (AT3G49120), *AtCAT1* (AT1G20630), *AtCBF1* (AT4G25490), *AtCBF2* (AT4G25470), *AtICE* (AT3G26744) and *AtCOR47* (AT1G20440). The expression level of *KoWRKY40* in transgenic lines was also measured. *Arabidopsis AtACTIN2* (AT3G18780) was used as a reference gene in the RT-qPCR reactions. The gene primer sequences used are listed in Supplemental Table S1. Relative gene expression values were calculated using the 2^-ΔΔCt^ Method as described above. Three biological replicates and three technical replicates were performed for each sample. The data was expressed as mean ± standard deviation (x ± SD). All the experiments were carried out in triplicate for biological replicates. Statistical analysis was performed with GraphPad Prism 7.0 (GraphPad Software, San Diego, California). One-way ANOVA followed by Duncan’s test was selected to quantify the significant differences by SPSS statistics 25.

## Supplementary Information

**Additional file 1: Supplementary Figure S1.** The phylogenetic analysis of KoWRKY40 with *Arabidopsis* WRKYs. The phylogenetic tree contained 58 *Arabidopsis thaliana* WRKYs, which were downloaded from the NCBI database and marked with accession numbers. The red triangle represented KoWRKY40 protein. The blue triangle represented AtWRKY1 protein, which was the template of KoWRKY40 for building 3D model. The scale indicates the length of the branch.

**Additional file 2: Supplementary Table S1** Gene primers used in real-time RT-PCR.

**Additional file 3: Supplementary Figure S2.** Original gel of Figure 6. RT-qPCR identification of *KoWRKY40* expression in WT and *KoWRKY40* transgenic lines. The *AtACTIN2* gene was amplified as a control.

## Data Availability

The sequence data of *KoWRKY40* can be obtained from the NCBI database with accession number: KP267757.1 (https://www.ncbi.nlm.nih.gov/nuccore/KP267757.1/). All analyzed or generated data is included in this article. The data analyzed or generated in this study can be obtained from the corresponding author with upon reasonable request.

## Abbreviations

TF: Transcription factor
GFP: Green fluorescent protein
RT-qPCR: Real-time quantitative PCR
DAPI: 4′,6-diamidino-2-phenylindole

## References

[1] Woodroffe CD, Rogers K, McKee KL, Lovelock CE, Mendelssohn I, Saintilan N (2016). Mangrove sedimentation and response to relative sea-level rise. Annu Rev Mar Sci.

[2] Muller C, Strydom NA (2017). Evidence for habitat residency and isotopic niche partitioning in a marine-estuarine-dependent species associated with mangrove habitats from the east coast of South Africa. Estuar Coast.

[3] Theuerkauff D, Rivera-Ingraham GA, Lambert S, Mercky Y, Lejeune M, Lignot JH, Sucré E (2020). Wastewater bioremediation by mangrove ecosystems impacts crab ecophysiology: in-situ caging experiment. Aquat Tox.

[4] Kathiresan K, Bingham BL (2001). Biology of mangroves and mangrove ecosystems. Adv Mar Biol.

[5] Chen J, Nolan T, Ye H, Zhang M, Tong H, Xin P, Chu J, Chu C, Li Z, Yin Y (2017). Arabidopsis WRKY46, WRKY54, and WRKY70 transcription factors are involved in brassinosteroid-regulated plant growth and drought responses. Plant Cell.

[6] Su WY, Ye CT, Zhang YH, Hao SQ, Li QS (2019). Identification of putative key genes for coastal environments and cold adaptation in mangrove *Kandelia obovata* through transcriptome analysis. Sci Total Environ.

[7] Baillo EH, Hanif MS, Guo Y, Zhang Z, Xu P, Algam SA (2020). Genome-wide identification of WRKY transcription factor family members in sorghum (*sorghum bicolor* (L.) moench). Plos One.

[8] Phukan UJ, Jeena GS, Shukla RK (2016). WRKY transcription factors: molecular regulation and stress responses in plants. Front Plant Sci.

[9] Huang S, Gao Y, Liu J, Peng X, Niu X, Fei Z, Cao S, Liu Y (2012). Genome-wide analysis of WRKY transcription factors in *Solanum lycopersicum*. Mol Genet Genomics.

[10] Xie Z, Zhang ZL, Zou X, Huang J, Ruas P, Thompson D, Shen QJ (2004). Annotations and functional analyses of the rice WRKY gene superfamily reveal positive and negative regulators of abscisic acid signaling in aleurone cells. Plant Physiol.

[11] Van Verk MC, Pappaioannou D, Neeleman L, Bol JF, Linthorst HJM (2008). A novel WRKY transcription factor is required for induction of PR-1a gene expression by salicylic acid and bacterial elicitors. Plant Physiol.

[12] Li FF, Zhang L, Ji HK, Xu ZY, Zhou Y, Yang SS (2020). The specific W-boxes of GAPC5 promoter bound by *TaWRKY* are involved in drought stress response in wheat. Plant Sci.

[13] Eulgem T, Rushton PJ, Robatzek S, Somssich IE (2000). The WRKY superfamily of plant transcription factors. Trends Plant Sci.

[14] Rushton PJ, Somssich IE, Ringler P, Shen QJ (2010). WRKY transcription factors. Trends Plant Sci.

[15] Mehtap SC, Gloria AM (2013). Identification of a drought- and cold-stress inducible WRKY gene in the cold-hardy *Citrus* relative *Poncirus trifoliate*. New Zeal J Crop Hort.

[16] Wang LN, Zhu W, Fang LC, Sun XM, Su LY, Liang ZC, Wang N, Londo JP, Li S, Xin H (2014). Genome-wide identification of WRKY family genes and their response to cold stress in *Vitis vinifera*. BMC Plant Biol.

[17] Wei ZP, Ye JF, Zhou ZQ, Chen F, Meng FJ, Liu YF (2021). Isolation and characterization of *PoWRKY*, an abiotic stress-related WRKY transcription factor from *Polygonatum odoratum*. Physiol Molbiol Pla.

[18] Zhu H, Jiang YN, Guo Y, Huang JB, Zhou MH, Tang YY, Sui JM, Wang JS, Qiao LX (2021). A novel salt inducible WRKY transcription factor gene, *AhWRKY75*, confers salt tolerance in transgenic peanut. Plant Physiol Bioch.

[19] Wang YJ, Jiang L, Chen JQ, Tao L, An Y, Cai HS, Guo CH (2018). Overexpression of the alfalfa WRKY11 gene enhances salt tolerance in soybean. Plos One.

[20] Wang Y, Shu Z, Wang W, Jiang X, Li D, Pan J, Li X (2016). *CsWRKY2*, a novel *WRKY* gene from *Camellia sinensis*, is involved in cold and drought stress responses. Biol Plantarum.

[21] Zhou QY, Tian AG, Zou HF, Xie ZM, Lei G, Huang J, Wang CM, Wang HW, Zhang JS, Chen SY (2008). Soybean WRKY-type transcription factor genes, *GmWRKY13*, *GmWRKY21*, and *GmWRKY54*, confer differential tolerance to abiotic stresses in transgenic *Arabidopsis* plants. Plant Biotechnol J.

[22] Peng YL, Wang YS, Fei J, Sun CC (2020). Isolation and expression analysis of two novel C-repeat binding factor (CBF) genes involved in plant growth and abiotic stress response in mangrove *Kandelia obovata*. Ecotoxicology.

[23] Fei J, Wang YS, Jiang ZY, Cheng H, Zhang JD (2015). Identification of cold tolerance genes from leaves of mangrove plant *Kandelia obovata* by suppression subtractive hybridization. Ecotoxicology.

[24] Dobson CM, Sali A, Karplus M (1998). Protein folding: a perspective from theory and experiment. Angew Chem Int Edit.

[25] Duan MR, Nan J, Liang YH, Mao P, Lu L, Li LF, Wei CH, Lai LH, Li Y, Su XD (2007). Arabidopsis thaliana DNA binding mechanism revealed by high resolution crystal structure of Arabidopsis thaliana WRKY1 protein. Nucleic Acids Res.

[26] Maeo K, Hayashi S, Kojima-Suzuki H, Morikami A, Nakamura K (2001). Role of conserved residues of the WRKY domain in the DNA-binding of tobacco WRKY family proteins. Biosci Biotechnol Biochem.

[27] Yamanaka T, Miyama M, Tada Y (2009). Transcriptome profiling of the mangrove plant *Bruguiera gymnorhiza* and identification of salt tolerance genes by *Agrobacterium* functional screening. Biosci Biotechnol Biochem.

[28] Eulgem T, Rushton PJ, Schmelzer E, Hahlbrock K, Somssich IE (1999). Early nuclear events in plant defence signalling: rapid gene activation by WRKY transcription factors. EMBO J.

[29] Chen LG, Song Y, Li SJ, Zhang LP, Zou CS, Yu DQ (2012). The role of WRKY transcription factors in plant abiotic stresses. Biochim Biophys Acta.

[30] Dong J, Chen C, Chen Z (2003). Expression profiles of the *Arabidopsis* WRKY gene superfamily during plant defense response. Plant Mol Biol.

[31] Ulker B, Somssich IE (2004). WRKY transcription factors: from DNA binding towards biological function. Curr Opin Plant Biol.

[32] Yamasaki K, Kigawa T, Inoue M, Tateno M, Yamasaki T, Yabuki T, Aoki M, Seki E, Matsuda T, Tomo Y, Hayami N, Terada T, Shirouzu M, Tanaka A, Seki M, Shinozaki K, Yokoyama S (2005). Solution structure of an Arabidopsis WRKY DNA binding domain. Plant Cell.

[33] Yang B, Jiang Y, Rahman MH, Deyholos MK, Kav NN (2009). Identification and expression analysis of WRKY transcription factor genes in canola (Brassica napus L.) in response to fungal pathogens and hormone treatments. BMC Plant Biol.

[34] Cheng Y, Yao Z, Ruan M, Ye Q, Wang R, Zhou G, et al. In silico identification and characterization of the WRKY gene superfamily in pepper (*Capsicum annuum* L.). Genet Mol Res. 2016;15:1–12. 10.4238/gmr.15038675 27706772

[35] Nuruzzaman M, Zhang R, Cao HZ, Luo ZY (2014). Plant pleiotropic drug resistance transporters: transport mechanism, gene expression, and function. J Integr Plant Biol.

[36] Agarwal P, Reddy M, Chikara J (2011). WRKY: its structure, evolutionary relationship, DNA-binding selectivity, role in stress tolerance and development of plants. Mol Biol Rep.

[37] Guo RY, Yu FF, Gao Z, An HL, Cao XC, Guo XQ (2011). *GhWRKY3*, a novel cotton (*Gossypium hirsutum* L.) WRKY gene, is involved in diverse stress responses. Mol Biol Rep.

[38] Yu XY, Pan Y, Dong Y, Lu B, Zhang C, Yang MS, Zuo LH (2021). Cloning and overexpression of *PeWRKY31* from *Populus* x *euramericana* enhances salt and biological tolerance in transgenic *Nicotiana*. BMC Plant Biol.

[39] Zheng LP, Yao JN, Gao FL, Chen L, Zhang C, Lian LL, Xie LY, Wu ZJ, Xie LH (2016). The subcellular localization and functional analysis of Fibrillarin2, a nucleolar protein in Nicotiana benthamiana. Biomed Res Int.

[40] Ma T, Li M, Zhao A, Xu X, Liu G, Cheng L (2014). *LcWRKY5*: an unknown function gene from sheepgrass improves drought tolerance in transgenic *Arabidopsis*. Plant Cell Rep.

[41] Jiang Y, Deyholos MK (2009). Functional characterization of *Arabidopsis* NaCl-inducible *WRKY25* and *WRKY33* transcription factors in abiotic stresses. Plant Mol Biol.

[42] Varagona MJ, Schmidt RJ, Raikhel NV (1992). Nuclear localization signal(s) required for nuclear targeting of the maize regulatory protein Opaque-2. Plant Cell.

[43] Yang L, Zhu H, Guo W, Zhang T (2010). Molecular cloning and characterization of five genes encoding pentatricopeptide repeat proteins from upland cotton (*Gossypium hirsutum* L.). Mol Biol Rep.

[44] Mare C, Mazzucotelli E, Crosatti C, Francia E, Stanca AM, Cattivelli L (2004). *Hv-WRKY38*: a new transcription factor involved in cold- and dehydration-response in barley. Plant Mol Biol.

[45] Rizhsky L, Liang H, Mittler R (2002). The combined effect of drought stress and heat shock on gene expression in tobacco. Plant Physiol.

[46] Ciolkowski I, Wanke D, Birkenbihl RP, Somssich IE (2008). Studies on DNA-binding selectivity of WRKY transcription factors lend structural clues into WRKY-domain function. Plant Mol Biol.

[47] Ezentgraf U, Laun T, Miao Y (2010). The complex regulation of *WRKY53* during leaf senescence of *Arabidopsis thaliana*. Eur J Cell Biol.

[48] Cai H, Yang S, Yan Y, Xiao Z, Cheng J, Wu J, Qiu A, Lai Y, Mou S, Guan D (2015). *CaWRKY6* transcriptionally activates *CaWRKY40*, regulates *Ralstonia solanacearum* resistance, and confers high-temperature and high-humidity tolerance in pepper. J Exp Bot.

[49] Zou C, Jiang W, Yu D (2010). Male gametophyte-specific *WRKY34* transcription factor mediates cold sensitivity of mature pollen in *Arabidopsis*. J Exp Bot.

[50] Du C, Zhao PP, Zhang HR, Li NN, Zheng LL, Wang YC (2017). The *Reaumuria trigyna* transcription factor *RtWRKY1* confers tolerance to salt stress in transgenic *Arabidopsis*. J Plant Physiol.

[51] Liu J, Zhu JK (1999). Proline accumulation and salt-stress induced gene expression in a salt-hypersensitive mutant of *Arabidopsis*. Plant Physiol.

[52] Armengaud P, Thiery L, Buhor N, Grenier-De March G, Savoure A (2004). Transcriptional regulation of proline biosynthesis in *Medicago truncatula* reveals developmental and environmental specific features. Physiol Plant.

[53] Liu K, Wang L, Xu Y, Chen N, Ma Q, Li F, Chong K (2007). Overexpression of OsCOIN, a putative cold inducible zinc finger protein, increased tolerance to chilling, salt and drought, and enhanced proline level in rice. Planta.

[54] Funck D, Winter G, Baumgarten L, Forlani G (2012). Requirement of proline synthesis during *Arabidopsis* reproductive development. BMC Plant Biol.

[55] Cecchini NM, Alvarez ME (2011). Proline dehydrogenase contributes to pathogen defense in *Arabidopsis*. Plant Physiol.

[56] Liu Y, Ji X, Nie X, Qu M, Zheng L, Tan Z, Zhao H, Huo L, Liu S, Zhang B, Wang Y (2015). Arabidopsis AtbHLH112 regulates the expression of genes involved inabiotic stress tolerance by binding to their E-box and GCG-box motifs. New Phytol.

[57] Zhang YT, Luo MW, Cheng LJ, Lin YX, Chen Q, Sun B, Gu XJ, Wang Y, Li MY, Luo Y, Wang XR, Zhang Y, Tang HR (2020). Identification of the cytosolic glucose-6-phosphate dehydrogenase gene from strawberry involved in cold stress response. Int J Mol Sci.

[58] Doğan M (2012). Investigation of the effect of salt stress on the antioxidant enzyme activities on the young and old leaves of salsola (*Stenoptera*) and tomato (*Lycopersicon esculentum* L.). African J Plant Sci.

[59] Shi W, Liu D, Hao LWC, Guo X, Li H (2014). *GhWRKY39*, a member of the WRKY transcription factor family in cotton, has a positive role in disease resistance and salt stress tolerance. Plant Cell Tissue Organ.

[60] Liu Q, Xu K, Pan Y, Jiang B, Liu G, Jia Y, Zhang H (2014). Functional analysis of a novel chrysanthemum WRKY transcription factor gene involved in salt tolerance. Plant Mol Biol Rep.

[61] Jaglo-Ottosen KR, Gilmour SJ, Zarka DG, Schabenberger O, Thomashow MF (1998). Arabidopsis CBF1 overexpression induces COR genes and enhances freezing tolerance. Science.

[62] Gilmour SJ, Sebolt AM, Salazar MP, Everard JD, Thomashow MF (2000). Overexpression of the *Arabidopsis CBF3* transcriptional activator mimics multiple biochemical changes associated with cold acclimation. Plant Physiol.

[63] Novillo F, Medina J, Salinas J (2007). *Arabidopsis CBF1* and *CBF3* have a different function than *CBF2* in cold acclimation and define different gene classes in the CBF regulon. Proc Natl Acad Sci U S A.

[64] Chinnusamy V, Zhu J, Zhu J (2007). Cold stress regulation of gene expression in plants. Trends Plant Sci.

[65] Miura K, Jin JB, Lee J, Yoo CY, Stirm V, Miura T, Ashworth EN, Bressan RA, Yun DJ, Hasegawa PM (2007). SIZ1-mediated sumoylation of ICE1 controls CBF3/DREB1A expression and freezing tolerance in *Arabidopsis*. Plant Cell.

[66] Fei J, Wang YS, Zhou Q, Gu JD (2015). Cloning and expression analysis of HSP70 gene from mangrove plant *Kandelia obovata* under cold stress. Ecotoxicology.

[67] Song H, Wang YS, Sun CC, Wang YT, Peng YL, Cheng H (2012). Effects of pyrene on antioxidant systems and lipid peroxidation level in mangrove plants, *Bruguiera gymnorrhiza*. Ecotoxicology.

[68] Bustin SA, Benes V, Garson JA, Hellemans J, Huggett J, Kubista M, Mueller R, Nolan T, Pfaffl MW, Shipley GL, Vandesompele J, Wittwer CT (2009). The MIQE guidelines: minimum information for publication of quantitative real-time PCR experiments. Clin Chem.

[69] Livak KJ, Schmittgen TD (2001). Analysis of relative gene expression data using real-time quantitative PCR and the 2(−Delta Delta C(T)) method. Methods.

[70] Bechtold N, Pelletier G (1998). In planta *Agrobacterium* mediated transformation of adult *Arabidopsis thaliana* plants by vacuum infiltration. Methods Mol Biol.

