# Supplementary material for: The Kandelia obovata transcription factor KoWRKY40 enhances cold tolerance in transgenic Arabidopsis
Source: BMC Plant Biol. 2022 Jun 4;22:274. doi: 10.1186/s12870-022-03661-2 (PMC9166612; doi:10.1186/s12870-022-03661-2)
Supplement: Supplementary file 1 — Additional file 1: Supplementary Figure S1. The phylogenetic analysis of KoWRKY40 with Arabidopsis WRKYs. The phylogenetic tree contained 58 Arabidopsis thaliana WRKYs, which were downloaded from the NCBI database and marked with accession numbers. The red triangle represented KoWRKY40 protein. The blue triangle represented AtWRKY1 protein, which was the template of KoWRKY40 for building 3D model. The scale indicates the length of the branch. [file 12870_2022_3661_MOESM1_ESM.docx]

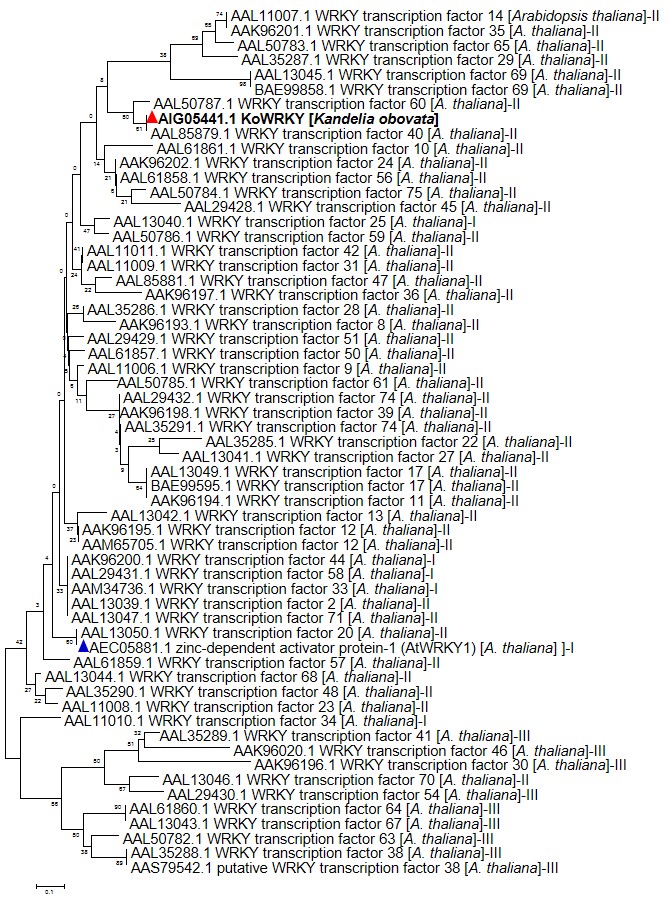


**Supplementary Figure S1. The phylogenetic analysis of KoWRKY40 with *Arabidopsis* WRKYs.** The phylogenetic tree contained 58 *Arabidopsis thaliana* WRKYs, which were downloaded from the NCBI database and marked with accession numbers. The red triangle represented KoWRKY40 protein. The blue triangle represented AtWRKY1 protein, which was the template of KoWRKY40 for building 3D model. The scale indicates the length of the branch.
