# Supplementary material for: The Kandelia obovata transcription factor KoWRKY40 enhances cold tolerance in transgenic Arabidopsis
Source: BMC Plant Biol. 2022 Jun 4;22:274. doi: 10.1186/s12870-022-03661-2 (PMC9166612; doi:10.1186/s12870-022-03661-2)
Supplement: Supplementary file 3 — Additional file 3: Supplementary Figure S2. Original gel of Figure 6. RT-qPCR identification of KoWRKY40 expression in WT and KoWRKY40 transgenic lines. The AtACTIN2 gene was amplified as a control. [file 12870_2022_3661_MOESM3_ESM.docx]

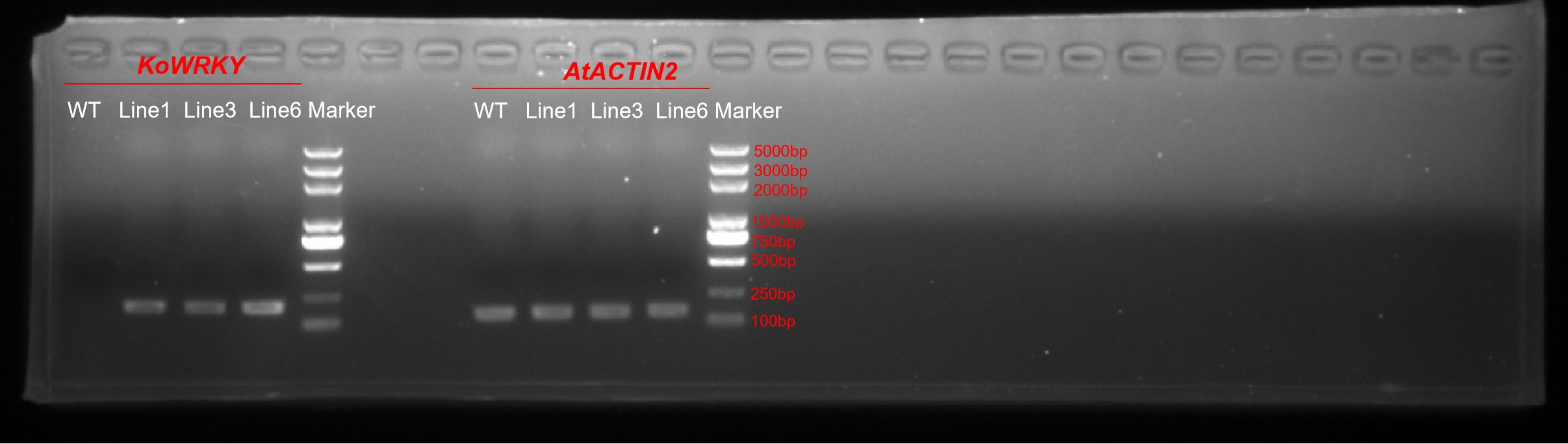


**Supplementary Figure S2.** Original gel of Figure 6. RT-qPCR identification of *KoWRKY40* expression in WT and *KoWRKY40* transgenic lines. The *AtACTIN2* gene was amplified as a control.
